# New biomarker: the gene HLA-DRA associated with low-grade glioma prognosis

**DOI:** 10.1186/s41016-022-00278-0

**Published:** 2022-05-19

**Authors:** Desheng Chen, Jiawei Yao, Bowen Hu, Liangwen Kuang, Binshun Xu, Haiyu Liu, Chao Dou, Guangzhi Wang, Mian Guo

**Affiliations:** Department of Neurosurgery, The Second Affiliated Hospital of Harbin Medical University, 246 Xuefu Road, Nangang, Harbin, 150086 Heilongjiang China

**Keywords:** Low-grade gliomas, HLA-DRA, Glioma, Immune, TEM, Biomarker

## Abstract

**Background:**

Low-grade gliomas (LGG) are WHO grade II tumors presenting as the most common primary malignant brain tumors in adults. Currently, LGG treatment involves either or a combination of surgery, radiation therapy, and chemotherapy. Despite the knowledge of constitutive genetic risk factors contributing to gliomas, the role of single genes as diagnostic and prognostic biomarkers is limited. The aim of the current study is to discover the predictive and prognostic genetic markers for LGG.

**Methods:**

Transcriptome data and clinical data were obtained from The Cancer Genome Atlas (TCGA) database. We first performed the tumor microenvironment (TME) survival analysis using the Kaplan-Meier method. An analysis was undertaken to screen for differentially expressed genes. The function of these genes was studied by Gene Ontology (GO) enrichment analysis and Kyoto Encyclopedia of Genes and Genomes (KEGG) pathway analysis. Following which a protein-protein interaction network (PPI) was constructed and visualized. Univariate and multivariate COX analyses were performed to obtain the probable prognostic genes. The key genes were selected by an intersection of core and prognostic genes. A clinical correlation analysis of single-gene expression was undertaken. GSEA enrichment analysis was performed to identify the function of key genes. Finally, a single gene-related correlation analysis was performed to identify the core immune cells involved in the development of LGG.

**Results:**

A total of 529 transcriptome data and 515 clinical samples were obtained from the TCGA. Immune cells and stromal cells were found to be significantly increased in the LGG microenvironment. The top five core genes intersected with the top 38 prognostically relevant genes and two key genes were identified. Our analysis revealed that a high expression of HLA-DRA was associated with a poor prognosis of LGG. Correlation analysis of immune cells showed that HLA-DRA expression level was related to immune infiltration, positively related to macrophage M1 phenotype, and negatively related to activation of NK cells.

**Conclusions:**

HLA-DRA may be an independent prognostic indicator and an important biomarker for diagnosing and predicting survival in LGG patients. It may also be associated with the immune infiltration phenotype in LGG.

## Background

Glioma is a common primary intracranial neuroepithelial tumor occurring in the brain and arising in glial tissue. Low-grade gliomas are generally referred to as WHO grade 3. Routine histopathological classification of gliomas [1]. However, the identification of key molecular alterations has led to substantial changes in the updated 2016 WHO Classification of Tumors of the CNS, such as IDH 1 and 2 mutations and 1p/19q codeletion [2, 3]. As such, the term “low-grade gliomas” is now often used to refer to both grade 2 and 3 gliomas, consistent with The Cancer Genome Atlas Project categorization. Molecularly altered gliomas are a heterogeneous group of primary brain tumors that vary not only in malignancy but also in histology and genomic alterations. They may arise from neural stem cells (NSCs), NSC-derived astrocytes, or oligodendrocyte precursor cells [4].

Low-grade gliomas are treated through surgery, chemotherapy, and/or radiation therapy. Despite the availability of therapeutic options, the possibility of recurrence exists [5]. Some of the factors that may contribute to ineffective treatment are the inability in getting around the blood-brain barrier for efficient drug delivery, overcoming immune-suppressive tumor microenvironment (TME), and development of drug resistance [6]. Significant advances have been made towards assessing the tumor microenvironment and have been used for treating LGG [7]. Recently, several immunotherapy methods have been shown to be successful in treating malignant tumors. Some of these include immune checkpoint blockade, cytokine therapy, cell therapy, and therapeutic vaccines [8]. But response rates vary between tumor types and within tumors. The variability in response limits the non-personalized use of immunotherapy for LGG [8]. The success of immunotherapy is thus dependent on a better understanding of the glioma-specific immune microenvironment [9]. While the genetic basis of gliomas is well-established, the utility of gene therapy in the field is less explored which can help overcome some of the therapeutic challenges and influence relapse rates in LGG. These gaps in the existing literature necessitate more research towards identifying new biomarkers and their applicability as therapeutic agents for LGG [10].

The major histocompatibility complex, class II, DR alpha (HLA-DRA) is a protein-coding gene. HLA-DRA is often associated with the occurrence of Graham-Little-Piccardi-Lassueur syndrome and penicillin allergy. The relevance of Parkinson’s disease with the HLA-DRA was also verified in a cohort of the Iranian population [11]. In addition, Lee et al. verified an intergenic variant rs9268877 between HLA-DRA and HLA-DRB contributing to the clinical course and long-term outcome of ulcerative colitis [12]. Chu et al. verified that HLA-DRA was a potential prognostic biomarker for renal clear cell carcinoma [13]. However, HLA-DRA has not been reported in LGG.

In this study, first, we explored the differential expression of HLA-DRA in LGG tissues. We correlated the expression of HLA-DRA and immune correlation in the LGG dataset of The Cancer Genome Atlas (TCGA) to ascertain the significance of HLA-DRA expression within the tumor samples. In addition, we performed GO, KEGG, COX, HPA, GSEA, and meta-analysis to assess the overall prognostic significance of HLA-DRA using data from TCGA databases and CGGA databases. Further, as substantial attention has been focused on the crucial role of the immune microenvironment in the progression of LGG [9, 14, 15], we also evaluated the potential correlation between HLA-DRA and immune infiltration levels in LGG by applying tools from the R software to data from the TCGA database.

## Methods

### Access to TCGA datasets

The gene transcriptome data tissues involving 529 samples and clinical data involving 515 samples were downloaded from The Cancer Genome Atlas (TCGA) database (https://portal.gdc.cancer.gov/) [16]. Clinical data collected included gender, age, grade, survival status, and duration of survival. Our study was in line with the publication guidelines provided by TCGA [17].

### Survival analysis of microenvironment

We compiled the transcriptome data using Perl (https://www.perl.org/). Similarly, the clinical data was summarized. ESTIMATE was used to assess the immune cell infiltration levels (measured as immune score) and stromal content (measured as stromal score) per sample. The aforementioned analysis was undertaken using the Limma and the Estimate package of the R software. Furthermore, microenvironmental survival analysis was performed using the survival package of the R software. The samples were grouped into high and low grade based on their median score. Meanwhile, the corresponding *p*-value and the number of patients at each time point were determined. The survival curve was plotted using the R software with the following screening criterion: adj *p* < 0.001. To verify the correlation between tumor microenvironment and LGG, we conducted a clinical correlation analysis of the microenvironment; the boxplot was plotted using the R package ggpubr.

### Differential analysis

Based on the ESTIMATE analysis, we sub-grouped patients as high and low, based on their immune and stromal score, respectively [15]. Differential expression analysis was performed between the high and low immune score groups and high and low stromal score groups by using the R package, with filter conditions of log2 |fold change| = 2 and false detection rate = 0.001. Heatmap and clustering were generated by using the “pheatmap” R package [15]. The intersection of differential gene results was obtained, and the Venn diagram of the differential gene was plotted. Subsequently, the clinical correlation was analyzed between stromal and immune cell scores.

### Gene Ontology and Kyoto Encyclopedia of Genes and Genomes enrichment analysis

We used the “ClusterProfiler” R package for functional enrichment analysis of differential genes to identify the potential functions and pathways [18]. Enrichment of genes was conducted by Gene Ontology (GO) and Kyoto Encyclopedia of Genes and Genomes (KEGG). *p*-value < 0.05 and *q*-value < 0.05 were considered as statistically significant.

### Protein–protein interaction (PPI) network

The PPI network was built using a search tool that retrieves a database of interacting genes (STRING) [19], and the minimum required interaction score was set to 0.95. We analyzed and visualized the network via Cytoscape software(https://cytoscape3.7.2.org/download.html) [20]. Network characteristics such as the number of core proteins the and interplay between different proteins were studied.

### COX regression model

Univariate and multivariate COX regression analysis and forest plots were plotted using the “forest plot” R package to display the *p*-values and hazard ratios (HRs) for each variable. Based on the results of COX analysis, prognostic-related genes were identified.

### Key gene acquisition

Through the intersection of PPI core genes and prognostic genes, we obtained the most probable key prognostic genes and plotted the Venn diagram of differential genes using the “VennDiagram” R package. To verify the prognostic correlation, we performed a survival analysis of the key gene by the survival package of the R software. LGG patients were divided into two groups of high and low expression according to the median value of gene expression, and the survival correlation was verified by comparing the postoperative differences between the high and low gene expression groups. The survival plot was drawn using “ggplot.” To verify the clinical relevance, the samples were divided into two groups according to age, gender, and grade, and the differences in the key gene expression levels were compared. A *p* < 0.05 was considered as statistically significant.

### Human Protein Atlas

The Human Protein Atlas (HPA, https://www.proteinatlas.org/) is a protein database that provides tissue and cellular distribution information for 26,000 human proteins [21]. In this study, we used the HPA to verify the HLA-DRA gene expression in glioma cells and glial brain cells.

### Gene set enrichment analysis

To explore the effects of the function or pathway of the gene expression in the tumor, samples were divided into two groups of high and low gene expression. Enrichment of the KEGG pathways in the high- and low-expression groups was further analyzed using the GSEA (ttp://software.broadinstitute.org/gsea/downloads.jsp) tool. The filter condition was set to 50, and the first 5 pathways were selected from the 50 enrichment pathways for multi-pathway enrichment analysis.

### Meta-analysis of the included studies

The RNA expression matrix and clinical data of low-grade glioma came from the TCGA database (ID: TCGALGG) and CGGA database (http://www.cgga.org.cn/, Dataset ID: mRNAseq_693 and mRNAseq_325). All data were corrected using the “limma” package in R. Clinical correlation analysis and univariate COX analysis were performed by the R. We got three COX files. Then, a meta-analysis was conducted using the R software to verify the correlation between the HLA-DRA gene and the prognosis of LGG. Heterogeneity was tested by *Q*-statistic and *I*^2^ statistic, *I*^2^ > 50% was considered as a significant heterogeneity, the random-effects model or the fixed-effects model was adopted, and *p* < 0.05 indicated that there were statistical significances.

### Immunological correlation analysis

The type and the relative number of immune cells in each tumor sample were visualized and plotted as a correlation curve by CIBERSORT [22]; the filter condition was set to a *p*-value = 0.05. Sample data with *p* < 0.05 were retained. Differential and correlation analyses were used to verify the correlation of immune cells and gene expression. The samples were divided into two groups of high and low gene expression. The presence of immune cells was comparatively analyzed between the two groups of high and low expression. The results obtained by the two analytical methods were intersected, to finally attain expressed immune cells in response to specific genes.

### Statistical analysis

All analyses were performed using the R software and Perl. A *p*-value < 0.05 was considered statistically significant.

## Results

### Tumor microenvironment with the prognosis of LGG

**Fig. 1:**
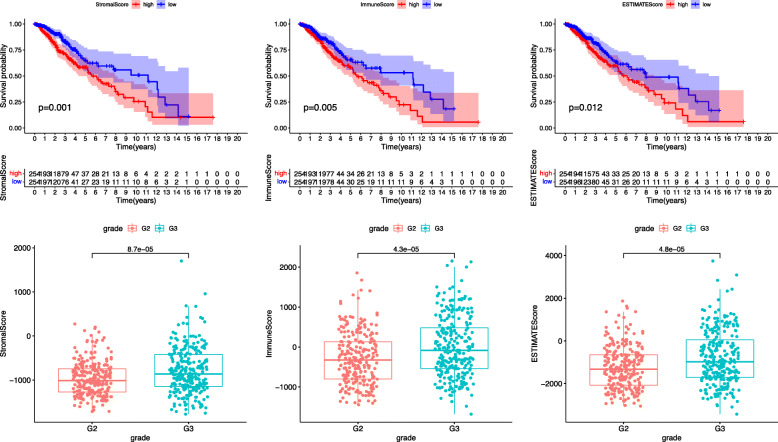
**A** Survival analysis of immune cells and stromal cells and comprehensive survival analysis. It is shown that the tumor microenvironment is associated with survival in LGG patients; the higher the content of stromal cells and immune cells, the worse the prognosis. *p* < 0.05 represents clinical significance. **B** The content of stromal cells and immune cells in the tumor microenvironment is independent of the patient’s age and sex and is related to the patient’s tumor grade. Patients presenting with grade III had higher levels of both

Transcriptome data (*n* = 529) and 515 clinical data extracted from the TCGA database were included in this study. An ESTIMATE analysis was performed. We found that the immune score of the selected samples was distributed between − 1681.2 and 2466.40, and the stromal score was distributed between − 1774.32 and 1701.59. By Kaplan-Meier analysis, we verified that the tumor microenvironment significantly correlated with survival in LGG patients (Fig. 1A). The clinical relevance of the microenvironment was also verified (Fig. 1B). As shown in the figure, the presence of stromal cells and immune cells in the tumor microenvironment is associated with the survival of the patient.

### Screening for differential genes

**Fig. 2:**
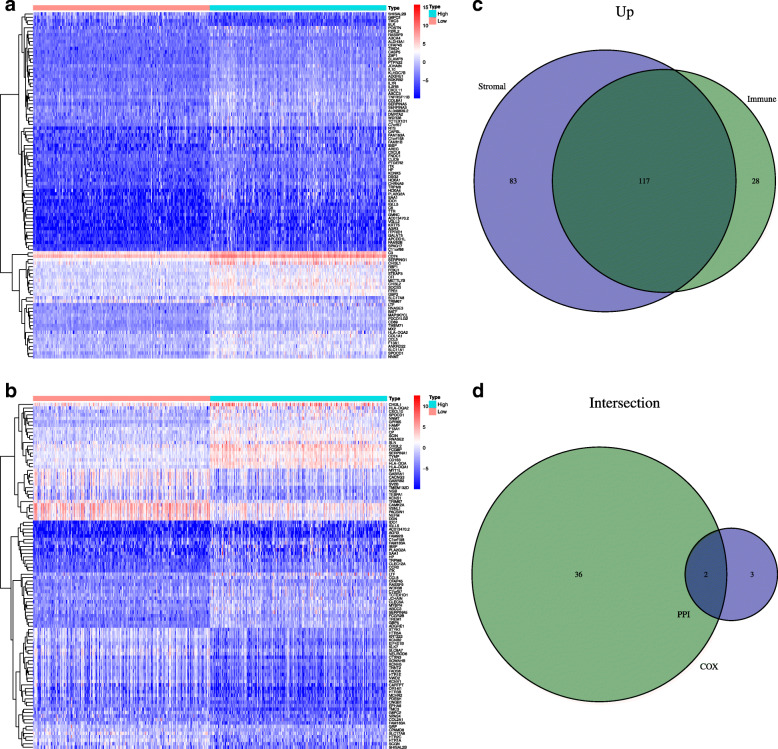
Differential analysis screens for differential genes. **A** Heat map of stromal cell difference genes. **B** Heat map of immune cell differential genes. Venn plot of the upregulated gene. **C** There are 117 intersecting genes. **D** The upregulation differential genes were visualized by protein interaction network analysis. COX analysis was performed for the differentially expressed genes. The intersection of the two analysis results is obtained. The intersection result is the core genes DLA-DRA and CD74

Differential analyses on stromal cells were performed to screen out upregulated genes and downregulated genes. Based on the comparative scores, we found 200 genes that were upregulated and 6 genes that were downregulated. Similarly, we performed a differential analysis on immune cells and found 145 genes that were upregulated and 65 genes that were downregulated. Following which we plotted a heatmap using the fold change and corrected *p*-value (Fig. 2A, B). The intersection of upregulated genomes was considered to ascertain differentially expressed genes. We found 117 genes that were upregulated (Fig. 2C).

### Functional enrichment analysis of differential genes

Upon undertaking a GO and KEGG enrichment analysis of the differentially expressed genes, we found that these genes were mainly responsible for T cell activation, leukocyte-mediated immunity, positive regulation of cytokine production, and cytokine−cytokine receptor interaction.

### Screening of hub gene

**Fig. 3:**
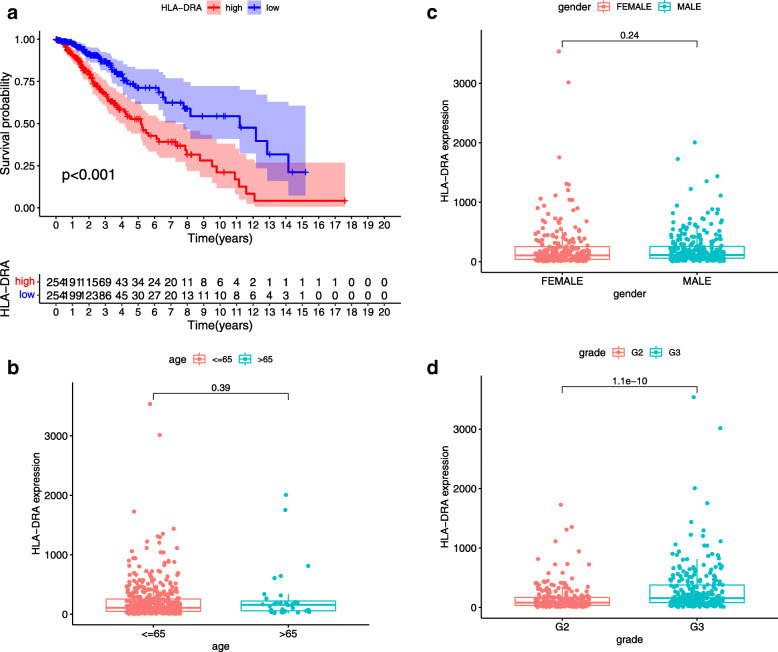
Single gene survival analysis and clinical correlation analysis were performed for gene HLA-DRA. **A** The results of survival analysis showed that HLA-DRA was correlated with the survival and prognosis of patients, and the prognosis of patients with high expression of HLA-DRA was worse. **B**–**D** Clinical correlation analysis showed that single gene expression was not correlated with gender and age, positive correlation with tumor grade

We obtained a protein interactome for the differential genes using the String database. The network was visualized and analyzed using the Cytoscape software. The analysis yielded 30 core genes. The COX analysis, performed on each sample, reveals 38 prognostically relevant genes. We took the top five genes with the most nodes in the PPI and the 38 genes associated with prognosis to build the intersection. The Venn diagram yielded two key genes HLA-DRA and CD74 (Fig. 2D). Since high expression of CD74 in gliomas has been validated for the association with poor prognosis and high immune infiltrates [23]. We proceeded with our analysis with HLA-DRA. Single-gene survival analysis showed *p* < 0.05, indicating that HLA-DRA gene expression was related to patient survival, and the high expression group predicted poor prognosis (Fig. 3A). Single-gene clinical correlation analysis showed that HLA-DRA expression was unrelated to age and gender. We found a positive correlation between the expression of HLA-DRA and LGG (Fig. 3B–D).

### Human Protein Atlas

**Fig. 4:**
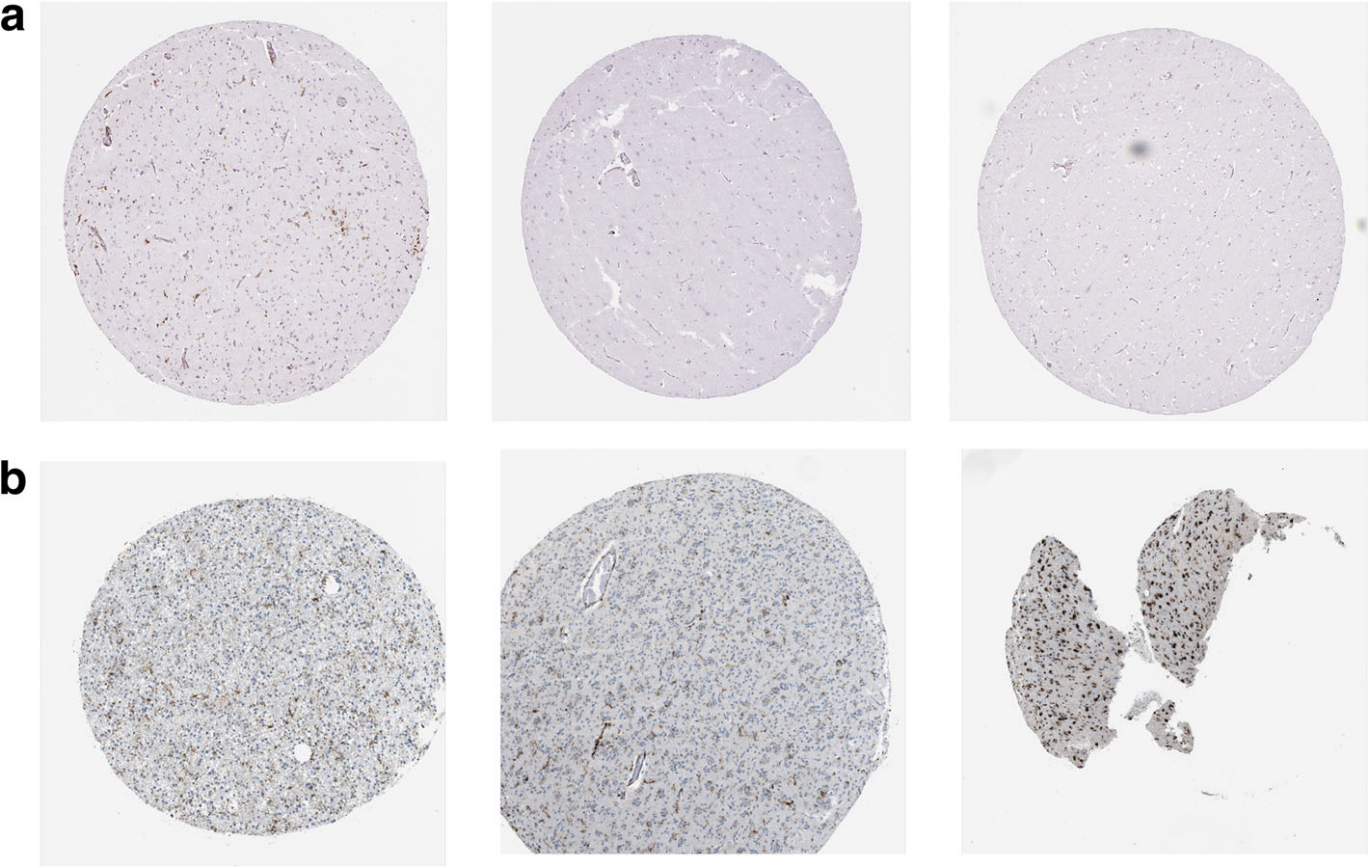
**A**, **B** Immunohistochemical analysis using HPA showed that the gene HLA-DRA was not expressed in early normal glioma cells (patient ID: 1371; 3731; 3739), but was highly expressed in glioma cells (patient ID: 3137; 3120; 3174)

To verify the differences in the HLA-DRA gene expression in tissues, we used the HPA to analyze the HLA-DRA gene expression in the glial cells and brain. We found that the HLA-DRA gene was not expressed in normal glial cells (patient ID: 1371; 3731; 3739) but was highly expressed in glioma cells (patient ID: 3137; 3120; 3174). The results of immunohistochemical staining were shown in Fig. 4A, B.

### Gene set enrichment analysis

The biological properties of HLA-DRA in LGG were analyzed by the GSEA enrichment method. The GSEA results showed that a high expression of HLA-DRA is mainly associated with cancer-associated cellular immunity whereas low expression is mainly associated with substance metabolism.

### Meta-analysis of the included studies

**Fig. 5:**
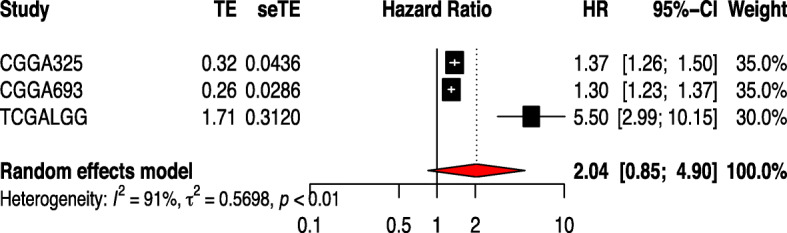
Clinical correlation analysis and univariate COX analysis were performed by the R. We got three COX files. Then, three COX documents were meta-analyzed by the meta package of R (*heterogeneity: I*^2^ = 91%, *p* < 0.01). The results show that HLA-DRA is highly uniform in the two databases. The reliability of the laboratory has been fully verified

Clinical correlation analysis and univariate COX analysis were performed by the R. We got three COX files. Then, three COX documents were meta-analyzed by the meta package of R. The meta-analysis results were shown in Fig. 5A.

### Gene-related immune cells

CIBERSORT [24] (http://cibersort.stanford.edu/) is a gene expression-based deconvolution algorithm that evaluates the expression changes of a group of genes relative to all other genes in the sample. Thus, the number of immune cells in each sample can be accurately enumerated through this process. The continued performance of CIBERSORT has prompted reliance on its utility to cellular heterogeneity research [25, 26]. Immune cell visualization and immune cell correlation analysis were performed by calculating the immune cell content by CIBERSORT. We found a differential expression of immune cells between the groups showing high and low gene expression. The correlation analysis verified the association between immune cell content and gene expression. Based on the differential analysis method, we found 12 types of immune cells. Based on the correlation analysis method, we found 9 types of immune cells. Of which we found an overlap of seven different immune cells, namely, plasma cells, activated natural killer (NK) cells, monocytes, macrophages M1 and M2 polarized, eosinophils, and activated mast cells.

## Discussion

Significant progress has been made in the treatment of glioma with postoperative chemotherapy, radiotherapy, and/or immune intervention, but the prognosis for patients with glioma continues to remain dismal. Over the past 30 years, epidemiological studies have shown that the overall survival rate of patients with glioma has been poor [27], and this seemingly has changed very little with advances in recent clinical practices involving immunotherapeutic approaches [28–30]. A better understanding of the tumor immune microenvironment is critical to improving the efficacy of current immunotherapies. Gene therapies have also been evaluated in preclinical and clinical settings [31, 32]. While off-target effects are a major concern for gene therapy, localized management of drugs and recently more advanced gene-editing tools have significantly increased the convenience and specificity of gene editing [33]. Therefore, there is still a need to accelerate research in the treatment of gliomas.

We used data from TCGA database and CGGA database and analyzed it using tools from the R language and Perl, to provide some verification for tumor microenvironment (TME) in low-grade glioma (LGG) and to identify key genes associated with prognostic immunity. First, we calculated the immune and stromal scores for each sample using the R software and found that the stromal cell and immune scores correlated significantly with LGG prognosis. The results showed that the stromal cells and immune cell scores of grade III gliomas were higher in comparison with grade I and II gliomas. This suggests that TME is associated with LGG tumor progression and disease prognosis.

Then, we performed a differential expression analysis on the high and low immune scoring groups and the stromal scoring group and identified 416 differentially expressed genes. Of which, 117 genes were found to be upregulated and six genes were found to be downregulated. The GO and KEGG analysis revealed that most of these genes were involved in the immune processes. The results show that immune cells and stromal cell in the LGG microenvironments are closely related which are concordant with previous findings [34, 35]. Through an R analysis, we obtained 123 related but differentially expressed genes. The protein–protein interactome (PPI) network explored the network relationship between survival genes and used Cytoscape for visualization. Significantly high network nodes, IL10, CCL2, CD74, and HLA-DRA, were found to be responsible for tumorigenesis, macrophage enrichment, and immune response among others [36–38]. Thirty-eight prognostic genes were obtained by the COX analysis, and then, the top five genes with the most nodes were selected from the PPI network. The intersection of the two genes was used to obtain the core gene HLA-RDA. Clinical correlation analysis of HLA-DRA showed that there was higher expression of HLA-DRA in grade III, which indicated that HLA-DRA could promote tumor development and serve as a reference for the utility of gene therapy in glioma. Then, we used the HPA to verify the HLA-DRA gene expression in glioma cells and glial brain cells. The results showed that HLA-DRA was not expressed in glioma cells but significantly expressed in glioma cells. This demonstrated the possibility of HLA-DRA as a new biomarker. The GSEA results showed that a high expression of HLA-DRA is mainly enriched in cancer-related cellular immunity whereas low expression is mainly enriched in substance metabolism. To better verify the reliability of the study, we adopted multiple databases for meta-analysis. The results show that HLA-DRA is highly uniform in the two databases. The results of immune cell correlation analysis showed that HLA-DRA was associated with a variety of immune cell infiltrates. This may indicate that HLA-DRA expression is correlated with multiple immune pathways or immune-related genes. At the same time, it provides a reference direction for future research.

From what has been discussed above, our results support the association of HLA-DRA with prognosis and immune infiltration of LGG. The mechanism of HLA-DRA in gliomas is not currently known, but our study validates the relevance of HLA-DRA to the tumor immune microenvironment. Further investigation into the association of HLA-DRA in the prognosis of LGG may allow to evaluate its utility as a potential therapeutic tool.

## Conclusions

In this study, we identified the gene HLA-DRA as an independent prognostic marker for LGG based on bioinformatics analysis. The results showed that with the occurrence of LGG, the content of stromal cells and immune cells in the microenvironment increased. The high expression of HLA-DRA is associated with the poor prognosis of LGG, and the high expression of HLA-DRA is closely related to the immune cells in the microenvironment.

## Data Availability

The original contributions presented in this study are contained in the article/supplementary material. Further inquiries may be made to the appropriate authors.
